# The Laryngeal Obtunder of Krishaber

**Published:** 1881-04

**Authors:** 


The Laryngeal Obtunder of Krishaber.—Tubage of the larynx, to which attention has been widely attracted during the past year by McEwan's method as a substitute in some cases for tracheotomy, has been put to what seems a more practical and important use by Krishaber in the method devised by him to prevent escape of blood into the trachea during bloody operations upon the nasal and buccal cavities. Upon the occasion of a recent operation for resection of the right superior maxillary bone, M. Verneuil, of Paris, demonstrated the use of the apparatus of Krishaba, and in a lecture based upon the case, strongly eulogized the method. This apparatus acts by obtunding the larynx, while still the patient is permitted to breathe, and that without tracheotomy or operation of any kind. It is composed of a bent tube, of proper length, having a diameter of eight millimetres, one of its ends being slightly widened. At four centimetres from the end which is to penetrate the larynx the tube is surrounded with a chemise of very thin rubber, perfectly sealed and communicating with a very small tube joined to the principal tube and terminating in a small stop-cock externally. In its application, the patient having been anesthetized, and the tongue drawn out by means of a forceps in the hands of an assistant, the operator touches with the left index finger the tip of the epiglottis; guided by this, he glides the end of the tube upon the finger-nail behind the epiglottis and into the larynx. That the instrument is well in place is known when the patient breathes through the tube. The rubber chemise is then dilated by water, injected by means of a syringe through the little tube communicating with it until the cavity of the larynx or trachea is completely filled. All communication around the tube is thus prevented, and respiration through the tube necessitated. Complete occlusion of the respiratory passages may be thus accomplished with facility. The experience of Krishaber has been very successful with it, and in the particular case where applied by M. Verneuil it acted perfectly. A moment's consideration will convince any one that the method is an important improvement. Simple, safe, thorough. The disadvantage of the position of Rose, the added dangers and difficulties of preliminary tracheotomy, and the use of the canula of Trendelenberg are avoided; continuous anesthesia is easily accomplished, and operations in the nasal, buccal, and pharyngeal spaces made as simple as in the extremities.—*Annals of Anat. and Surg.*

